# Neurobehavioral Mechanisms of Sodium Appetite

**DOI:** 10.3390/nu15030620

**Published:** 2023-01-25

**Authors:** Neil E. Rowland

**Affiliations:** Department of Psychology, University of Florida, Gainesville, FL 32611-2250, USA; nrowland@ufl.edu

**Keywords:** sodium appetite, sodium deficiency, satiation, aldosterone, angiotensin, nucleus of the solitary tract, sodium deficient diet, optogenetic, chemogenetic

## Abstract

The objectives of this paper are to first present physiological and ecological aspects of the unique motivational state of sodium appetite, then to focus on systemic physiology and brain mechanisms. I describe how laboratory protocols have been developed to allow the study of sodium appetite under controlled conditions, and focus on two such conditions specifically. The first of these is the presentation a sodium-deficient diet (SDD) for at least one week, and the second is accelerated sodium loss using SDD for 1–2 days coupled with the diuretic furosemide. The modality of consumption is also considered, ranging from a free intake of high concentration of sodium solution, to sodium-rich food or gels, and to operant protocols. I describe the pivotal role of angiotensin and aldosterone in these appetites and discuss whether the intakes or appetite are matched to the physiological need state. Several brain systems have been identified, most recently and microscopically using molecular biological methods. These include clusters in both the hindbrain and the forebrain. Satiation of sodium appetite is often studied using concentrated sodium solutions, but these can be consumed in apparent excess, and I suggest that future studies of satiation might emulate natural conditions in which excess consumption does not occur, using either SDD only as a stimulus, offering a sodium-rich food for the assessment of appetite, or a simple operant task.

## 1. Introduction

### 1.1. Sodium Homeostasis

In biological systems, the cation sodium is always paired with an anion. This anion is predominantly chloride. In this paper, unless noted, the term sodium implies sodium chloride. Sodium is the principal cation of the ECF compartment. In most animals, including humans, the concentration of sodium in the ECF is approximately 140 mOsm/kg. In contrast, the concentration of sodium in the ICF is much less, typically about 5 mOsm/kg. This concentration gradient is maintained by a plethora of ion channels, e.g., sodium channels and aquaporins, and sodium pumps spanning cell membranes [1]. If the concentration of sodium in ECF increases, water is drawn osmotically from the ICF to the ECF with a resultant shrinkage of cells. The processes governing this are collectively known as osmoregulation, and cells that transduce that change into neural signals in or to the brain are osmoreceptors [2]. These receptors underlie what is known as osmotic thirst which is functionally a specific appetite for water.

Sodium has a pivotal role in maintaining the volume(s) of the body fluid compartments and this is accomplished via accurate and often redundant mechanisms to achieve constancy or near constancy of sodium content and distribution. Terrestrial animals continuously lose sodium to their environment via urine and sweat, and to a smaller extent in feces. The amount of sodium so lost will be the product of the volume and sodium concentration of the excreted fluids.

Mammalian kidneys are composed of nephrons (see [3] for a recent and more detailed review) which operate on a perfusion-reabsorption principle. Humans have one million nephrons in each kidney. Over 99% of the ECF filtered from blood by the nephron is recovered and that fraction is modulated by the action of hormones, including aldosterone and ADH, which increase sodium and water reabsorption, respectively, in the distal tubule. The unrecovered fluid becomes urine. A high ratio of potassium to sodium in urine can be used as a non-invasive marker of elevated blood aldosterone.

Sodium depletion is associated with a large increase in plasma aldosterone concentrations, achieved by activation of the enzyme aldosterone synthase in the adrenal cortex. Factors that activate aldosterone synthase include ANG II, ACTH, and plasma potassium concentration. ANG II is formed by successive cleavage of angiotensinogen to the decapeptide ANG I by renin and to the octapeptide ANG II by angiotensin-converting enzyme type 1. Angiotensinogen is synthesized in both the liver and brain. The activity of the peripheral system often is assessed using PRA which is elevated in sodium deficiency (see [4] for more comprehensive review).

Humans may also lose substantial fluid and sodium through sweating (see [5] for recent overview). Eccrine cells which mediate sweating are particularly abundant on the forehead and hands of humans. Sweat is produced by ion transport and water movement from blood plasma in each sweat gland. The concentration of sodium in sweat decreases as blood levels of aldosterone increase. The volume of sweat produced per unit time is limited by the diameter of the sweat duct which is controlled by the sympathetic nervous system. The sweat rate is high in hot environments due to evaporative cooling, or when endogenous heat production is high, such as during exercise. In some species, including rats and mice, saliva which is normally swallowed is spread on the fur to accomplish evaporative cooling in place of sweating.

### 1.2. Sodium Preference and Sodium Appetite

This is a paper about sodium appetite, mostly in laboratory animals. It is important to clearly articulate what is meant by the term sodium appetite, at least in animals. Sodium appetite may be defined operationally as consumption of significant amounts of sodium (in any form) that normally would not be consumed, and due to a defined physiological manipulation or state. In contrast, sodium preference is voluntary consumption of sodium in its relevant vehicle without known state or manipulation to cause that consumption. Preference may be assessed by presenting two (or more) versions of the vehicle, only one of which contains sodium, and observing that a significant fraction is taken from the sodium containing version [6]. The most natural state that induces sodium appetite is hypovolemia which entails loss of sodium containing ECF. In hypovolemia, thirst may occur with or before sodium appetite [7].

Most laboratory studies of sodium intake use aqueous solutions (i.e., water is the vehicle), but the definition above does not require any specific physical form. Sodium intake can be assessed by measuring the intake of a single solution of NaCl or it can be assessed using two (or more) bottle intakes, one of which is water and another NaCl of the test concentration. The test may be brief (seconds or minutes) or long (up to 24 h). These durations may emphasize pre- and post-absorptive factors, respectively. Because most spontaneous fluid ingestion in rodents occurs as small bouts throughout the night [1], measurements of intake are often made over periods of 3–24 h, but this duration guarantees postabsorptive impact of the ingested sodium. To circumvent this aspect, some studies have used brief access to solutions (typically several are tested contiguously) and the number of licks emitted per solution are recorded and compared. This reflects a pure taste reactivity. Many but not all strains of non-depleted mice and rats have, in a choice with water, a high spontaneous preference for slightly hypotonic (<0.15 M) NaCl solutions over water, and a low preference at concentrations well above isotonic (e.g., >0.2 M).

### 1.3. Sodium in the Environment

At the start of his monograph “The Hunger for Salt” [8], Denton states:

*“In large areas of the planet, as a result of meteorological conditions, there is very little sodium. Accordingly, for a wide diversity of animal species in different ecological niches, there is great survival value in the possession of effective mechanisms for acquisition of salt, and of endocrine mechanisms for its retention in the body”*

(Denton, 1982, p1)

Denton later points out that many terrestrial species, including our human ancestors, live in a condition of chronic mild sodium insufficiency precisely because the environment does not contain enough sodium to ensure eunatremia. The endocrine mechanisms for retaining sodium are very efficient but cannot reduce daily loss to zero. Hence, net sodium lost must be replaced by sodium intake and for terrestrial animals this must occur through periodic salt seeking and ingestion. Water in the environment is not a reliable option: water from rain, rivers, and lakes usually contains negligible amounts of sodium, while brackish water from wells or oases (or from mineral deposits, i.e., salt licks) is geographically infrequent. Instead, sodium may normally be gleaned from food. Most plants have low sodium content, determined in part by the concentration of sodium in the ground water which is highest near the oceans and lowest far inland and/or at altitude [8], p 12. However, a few plants are sodium concentrators and are sought after. In contrast, animal source foods are rich in sodium; omnivores typically ingest adequate sufficient sodium in the mixture of foods they eat.

Rodgers [9] showed that rats depleted of sodium by feeding a sodium deficient diet for several weeks subsequently presented a short-term preference for a sodium-containing diet, and that this was dependent on their tasting the salt. This finding was extended in a comprehensive examination of dietary preference in rats after acute sodium depletion by 48 h of sodium deficient diet (SDD) and injection of furosemide (which induces acute diuresis and natriuresis) after 24 h [10]. Furosemide induces acute natriuresis and diuresis, with excretion of near-isotonic urine for 1–2 h (in rats, about 2 mEq) and thereafter for the remainder of the 24 h little additional sodium is lost [11]. In one of Bertino and Tordoff’s experiments [10], depleted or non-depleted rats were given a four-day choice between SDD and the same diet containing either 0.5, 1, 2, 4, or 8% NaCl. Data for the first 2 h of the diet choice (Table 1) showed a strong preference for the salted food even at the high concentration of 8%. In contrast, non-depleted rats showed a net avoidance of the salty food, more so at the higher concentrations. The authors make the point that, even at the lowest concentrations, this dietary preference must be driven by a perceived taste of salt. Indeed, it is well-known that sodium appetite is dependent on the specific taste of sodium [8]. Studies in which ingestion bypasses the oral cavity, and/or using an inhibitor (e.g., amiloride) of the lingual sodium receptors, reveal a reduced or abolished sodium appetite. Further, cations such as potassium are rejected by sodium-depleted animals.

Although sodium in food, and at relatively low concentrations, may be the natural way in which many animals manage their sodium balance, most laboratory tests use fluids. Such tests often offer a choice between water and hypertonic NaCl. Depending on species and strain, sodium solutions above approximately 0.3 M are not preferred and functionally provide an example of an obstacle to be overcome, which is a benchmark for motivated behavior. Taste adulteration [12] or operant response costs [13] have been used and will be mentioned below.

### 1.4. Procedures That Induce Sodium Appetite

In this section, I will explain the characteristics of the laboratory procedures commonly used to induce sodium appetite in rodents. These are a subset of many procedures that have been shown to induce sodium appetite (see [6] for review). From the foregoing sections, it may be deduced that the natural stimulus of sodium appetite is a suitably long period of low sodium diet. In laboratory rodents fed a sodium-free diet for up to seven days at normal ambient temperatures, the resulting sodium appetite from a concentrated sodium solution is typically small but sufficient to restore the physiological deficit [11,14]. Some of the studies of brain mechanism to be discussed later in this paper have used such a natural depletion, but it is demanding in the sense that cages need to be cleaned and bedding changed frequently to assure the environment does not contain urine or other materials that may contain sodium.

Instead, a briefer procedure for depletion [11] has become the standard even though, as I will describe, some aspects of the results are not the same as for the longer procedure. The rapid procedure applied to rats and mice involves feeding a very low sodium (usually purified ingredient) diet, hereafter referred to as sodium deficient diet (SDD), for at least 24 h following one or more injections of furosemide [11,15]. Water is usually available during the 24 h depletion period to allow satisfaction of thirst. At the end of this time, the subjects are presented with sodium solution and water, and intakes are measured at appropriate intervals thereafter. The induced sodium appetite is typically complete within 1 h.

The time course for development of sodium appetite in this protocol in female rats was reported by Rowland & Morian [16]. In separate groups, intake of 0.3 M NaCl as well as select physiological parameters were measured 3, 12, or 24 h after furosemide. The timing of the furosemide injections was such that the tests and measures were all made at the same time of day (early afternoon). Some of the principal results are shown in Table 2. First, despite the fact all sodium is lost in the first 1–2 h [11], the sodium appetite was modest after 3 h and took 24 h to fully mature. It should be noted that the intake after 12 h approximates the typical deficit accrued after this treatment, but the intake after 24 h is manifestly greater than the need. Second, while aldosterone continued to rise for 12 h, PRA was maximal after 3 h and declined thereafter. Third, plasma volume as indexed by protein concentration was maximally reduced after 3 h and then recovered partially. Fourth, Fos-ir (an index of neuronal activation) in SFO and OVLT was elevated comparably at all times (see Section 2 for further examples and discussion of Fos studies). No one, or simple combination, of these variables correlates with the amount of sodium consumed. One resolution of the time course issues would be the existence of a slow-depleting and -repleting sodium reservoir. While the older literature failed to identify such a reservoir—please see [8] and [12] for citations and discussion—, the skin [17] and bone [18] have now emerged as viable candidates.

Jalowiec [11] detailed the sodium losses and intakes in four groups of rats. Two of the groups were maintained on sodium replete diet (chow) while the other two groups were placed on SDD for six days. All groups had access to water and 0.51 M NaCl during this phase of the study. Despite having access to salt during this run-in period, the SDD groups remained in a slightly negative sodium balance (−0.45 mEq) and had a high urinary K/Na ratio of approximately 100, indicative of elevated aldosterone. On the 7th day, half of the rats in each group received furosemide (10 mg) and the other half received vehicle. All groups were then deprived of food and 0.51 M NaCl but not water for the next 24 h. At the end of this time, during which urinary sodium loss was measured, access to 0.51 M NaCl was restored and intakes measured for 3 h. The key results are shown in Table 3. As expected, rats previously fed chow and injected with vehicle lost a small amount of sodium in urine over 24 h (due to the food restriction) but consumed very little NaCl in 1 h so tolerating the deficit of about 0.8 mEq. Notice that the deficit of the SDD groups during the run-in period was less than this, and it too was not offset by increased NaCl consumption, suggesting that deficits of less than approximately 1 mEq (3 mEq/kg when adjusted for body weight) are in a tolerated range. The previously chow fed group given furosemide lost the most sodium (2.6 mEq) but drank only enough NaCl to come close to the tolerated range of deficit as described above. The SDD maintained group given vehicle sustained little additional sodium loss and drank a small amount of NaCl which yielded a small net positive sodium balance that was close to the average sodium lost during the run-in period. Finally, the SDD group treated with furosemide showed a strong natriuresis (1.5 mEq), albeit less than the chow-furosemide group presumably because of their initially depleted status. Nonetheless, they showed the greatest sodium intake (3.5 mEq, or about 7 mL of the 0.51 M solution available) and this took them into a strong positive sodium balance. That is, they consumed more salt than the measured loss, a finding that has been noted by investigators using variations of this protocol, including a shorter run-in period (0 or one day) and with SDD available during the 24 h depletion period (e.g., [16]. The reason for this apparently excessive consumption is not known, but it is important to note this does not occur in furosemide-treated rats previously on chow [11] or after many days of SDD without furosemide [9,11]. It also does not occur in SDD-furosemide rats given mildly unpalatable (pH = 12) 0.3 M NaCl with an intake of 5.2 mL or 1.6 mEq [12], in rats given dilute (0.03 M) NaCl to drink [19], or in SDD-chronic furosemide rats working for either 0.3 M or 0.45 M NaCl reinforcements on progressive ratio schedules with intakes of approximately 1.7 and 1.8 mEq, respectively, compared with 3–4 mEq when free drinking [20].

Rowland, Farnbauch & Crews [14] examined sodium loss and appetite in albino mice following depletion protocols adapted from Jalowiec [11]. Mice were fed SDD for either one or seven days prior to injection of vehicle or furosemide (2 × 40 mg/kg or about 4 mg/mouse) and provided water and 0.15 M NaCl 24 h later. Other mice had blood samples collected and these were comparable to or slightly greater than corresponding values of aldosterone, PRA, and plasma protein in depleted rats. Furosemide-treated groups lost approximately 1.5 g body weight above that of the vehicle controls, indicative of a urinary loss (assuming isotonicity) of 0.22 mEq which transforms to ~5 mEq/kg. This is comparable to the weight-adjusted loss using similar protocols in rats. The 2-h intake of 0.15 M NaCl in the seven-day SDD group (0.5 mL) was not above that of non-depleted controls, whereas intakes of both one- and seven-day SDD groups treated with furosemide were approximately 2.2 mL (0.33 mEq). These are some 50% higher than the estimated deficit. Other furosemide-treated SDD mice received 0.5 M NaCl solution and consumed about 1.0 mL (0.5 mEq). Thus, the mice consumed more than their loss by about 50% and 100% when presented with 0.15 M or 0.5 M NaCl, respectively. The latter is similar to the high consumption of hypertonic NaCl by depleted rats. To determine whether the solution form of NaCl is critical, other mice were chronically treated with SDD and furosemide and could replete their recurring deficit for 2 h/day by consuming a gel matrix containing either 0.5, 1.0, or 1.5 M NaCl. The gel intakes were linearly negatively correlated with concentration, netting about 0.3 mEq sodium per day. This amount is approximately half that consumed from 0.5 M NaCl solution but was sufficient to maintain body weight (presumably by meeting physiological need) in this chronic protocol. Thus, concentrated NaCl solution is unique in producing overconsumption in the SDD-furosemide protocols. This finding is relevant to the interpretation of mechanistic studies of satiation of sodium appetite to be described later.

It was mentioned earlier that the taste of sodium is essential for sodium appetite. It follows that untasted sodium may be relatively ineffective in satiating sodium appetite. In this regard, the finding of Wolf, Schulkin & Simson [12] is particularly clear. Rats were depleted of sodium using an SDD-furosemide protocol and were then given NaCl (5 mL of 3% NaCl or 2.5 mEq, at least equal to their physiological deficit) either orally or by gastric gavage at intervals of 0.5–16 h prior to a sodium consumption test. At this test, rats with prior oral loads consumed approximately 2 mL 0.3 M (pH12) saline, regardless of the preload-to-test interval, whereas those given the preload intragastrically showed a smaller satiation at intervals up to 4 h (intakes ~3 mL, still less than the 5 mL intake of non-repleted controls) and approached the efficacy of oral preloads only after a delay of 16 h.

## 2. Materials and Methods

### 2.1. Brain Mechanisms and Sodium Appetite

It is well established that the structures along the lamina terminalis of the midline third cerebral ventricle, namely the SFO, MnPO, and OVLT, are involved in thirst. Further, peripheral detection of hormones such as ANG II by the area postrema engages the NST which projects indirectly via the pontine lateral PBN to the lamina terminalis and other structures. Traditional approaches to elucidating the structures and pathways for sodium appetite have often been based on the “dual depletion” model according to which aldosterone and ANG II act synergistically to induce sodium appetite [21,22]. Some studies, including lesions and induction of Fos-ir implicated the lamina terminalis, ostensibly the site(s) for ANG II transduction, but those for aldosterone were not known.

Lesions of the SFO and OVLT decrease sodium appetite after depletion [23,24,25] but see [26], findings that are consistent with the hypothesis that the transduction sites for ANG II in sodium appetite are located in these structures of the lamina terminalis. The involvement of forebrain structures, including the lamina terminalis, is consistent with the finding that complete brainstem transection in rats abolishes intake of and oromotor taste reactivity to intraorally delivered salt solutions following furosemide-induced depletion [27]. Other structures that were implicated include parts of the limbic system and hypothalamus (see [8] for review). Since that time, dopamine and/or dynorphin in the striatum and/or nucleus accumbens have been implicated [28,29,30]. Further, repeated sodium depletion using the SDD-furosemide model which, in some studies potentiate the already excessive sodium intake [31,32], increases dendritic branching in medium spiny neurons of the nucleus accumbens [32]. However, there was no clear integration among these disparate findings. Over the past 20 years, the advent of molecular biological methods has greatly altered the landscape, as will be reviewed in the following sections.

### 2.2. Hindbrain Mechanisms

Corticosterone, which circulates in much greater concentrations than aldosterone, has an equal affinity for the so-called mineralocorticoid receptor in the nucleus. The specificity of action of aldosterone at this receptor is enabled by co-expression of the enzyme 11β-HSD2, which converts corticosterone to inactive cortisone, leaving aldosterone to act at its receptor. The key observation relative to brain function was the discovery that a population of neurons in rat NST express 11β-HSD2 [33]. Using Fos-ir as a metric of activation, Geerling et al. [34] then showed that these neurons were engaged progressively by feeding SDD, with induction of Fos-ir peaking at about 35% of HSD2 cells after six days. Further, this Fos-ir was completely reversed within 2 h after access to 0.3 M NaCl solution. These authors also reported comparable activation of HSD2 neurons after 48 h of SSD coupled with furosemide. It should be noted that adrenally produced steroids are not essential for activation of the HSD2 neurons in rats: adrenalectomized rats given SSD for just 24 h showed the same or slightly greater fraction of Fos-positive HSD2 cells as intact rats deprived of sodium for seven days [34]. This suggests that other signals of sodium depletion, including possibly ANG II or signal(s) from the various sodium reservoirs, are capable of activating the HSD2 cells in NST.

Using rats, Formenti et al. [35] found that chronic infusion of aldosterone via cannula into the 4th cerebral ventricle, but not into the lateral (anterior) ventricle, stimulated sodium intake. At the higher dose used (100 ng/h), the 24 h intake of 0.3 M NaCl increase to an extraordinary mean of 130 mL. Conversely, acute 4th ventricular administration of a mineralocorticoid receptor antagonist attenuated sodium intake after furosemide depletion. The proximity of the NST and 4th ventricle strongly suggests that the HSD2-expressing cells are responsible for these bidirectional effects on sodium appetite.

Additional support for this assertion was provided by Jarvie & Palmiter [36] who generated mice with Cre recombinase targeted to the gene encoding HSD2 (*Hsd11b2*). The NST was then injected with an AAV construct that would enable expression of the excitatory G-protein coupled designer receptor hM3Dq in the HSD2 neurons. Subsequently, peripheral injection of CNO was used to activate these transfected receptors for a few hours. In a two-bottle choice with water, the hM3Dq mice consumed a mean of 1.5 mL 0.5 M NaCl in a 4-h test at the start of the night phase, compared with ~0.2 mL in the controls. This intake is comparable to that observed 24 h after SDD-furosemide depletion by these and other investigators. The next night, without CNO, intake of these mice had returned to normal, indicating no long-term effects of this degree of HSD2 stimulation on sodium preference.

Specific chemogenetic inhibition of HSD2 neurons using the inhibitory hM4Di receptor constructs only a partially inhibited sodium appetite of mice depleted with furosemide [36], similar to the result with the mineralocorticoid antagonist in rats [35]. It is likely that this is because ANG II itself has a stimulating effect on sodium appetite in sodium depletion (see below), but normally acts together with aldosterone. Palmiter & Jarvie [36] also showed that the principal projection areas or downstream targets of the HSD2 cells in NST were the mediolateral part of the PBN, the pre-LC, and the forebrain ventral division of the BNST. Fos-ir was induced in each of these regions after chemogenetic stimulation of the HSD2 cells (via hM3Dq and CNO), or by sodium depletion in wild type mice. In lateral PBN and pre-LC about 40% of these Fos-ir cells were also immunopositive for the transcription factor Foxp2.

Resch et al. [37] examined electrophysiological properties of HSD2-expressing neurons of NTS using slice preparations. To visualize these neurons, *Hsd11b2 Cre* knockin mice were first crossed with Ai9tdTomato mice. The result was near-complete co-localization of HSD2 with tdTomato. These mice were sodium depleted either by several days of SDD or accelerated by furosemide, as described previously. The HSD2 neurons in control mice had low firing rates (<0.5 Hz) while those in sodium-depleted mice had an approximately five-fold increase in rate of firing. Direct application of aldosterone to the slices had no effect, but in vivo pretreatment with aldosterone via minipump for approximately 10 days increased the firing rate comparably to SDD.

From single cell mRNA sequencing, Resch et al. [37] determined that the NTS-HSD cells express high levels of the ANG II receptor (AT1R) mRNA. They then showed that adding ANG II to the medium bathing the slice preparations from non-depleted mice increased the firing rate, an effect that was blocked by the AT1R antagonist losartan. The elevated firing rate in cells from mice treated in vivo with aldosterone was further increased by bath application of ANG II. These neurons thus are co-responsive to ANGII and aldosterone in an apparent additive manner.

Next, Resch et al. [37] determined that the HSD2 cells are glutamatergic and thus generate EPSPs in their terminal fields. To determine which of the projection fields might be involved in sodium appetite, AAV-FLEX-ChR2 was injected into the NST of *Hsd11b2-Cre* mice and, after allowing time for anterograde transport, optic fibers were implanted over either the PBN or ventral BNST. Laser stimulation of ChR2 in ventral BNST, but not PBN, induced licking for sodium. In a later section of this paper, we will describe projections from SFO to the same region of the BNST that also have been implicated in sodium appetite [38].

### 2.3. Forebrain Mechanisms

Acute injection or chronic infusion of ANG II into the anterior cerebral ventricles of rats is dipsogenic, but also potentiates intake of sodium solutions [22]. In rats with sodium appetite induced by furosemide-deficient diet, lateral ventricular injection of the AT1R antagonist losartan had no effect on sodium intake [39]. As noted previously, some but not all SFO lesion studies in rats impair sodium appetite. Sodium depletion with either furosemide or peritoneal dialysis rapidly induces Fos-ir in SFO and dorsal cap of the OVLT, but not in the neurosecretory regions, i.e., supraoptic nuclei and PVN in rats and mice [40,41,42,43]. Crews & Rowland [43] showed that both the sodium appetite and the Fos-ir in SFO and OVLT observed 24 h after furosemide were inhibited by subcutaneous pretreatment (1 h before test) with losartan. Because Fos-ir is maximal in SFO 3 h after furosemide [16] but sodium appetite is not induced until later and is then reversed by losartan may indicate that different subpopulations of cells in the SFO are implicated at different times.

This issue has been addressed in studies by Matsuda et al. [38]. C57BL/6J mice, manipulated as will be described, were studied using a single injection of furosemide (400 mg/kg) followed by 3 h without food or water (water and sodium depleted condition). Other mice received furosemide and were presented with SDD and water for 24 h (sodium-depleted condition). In a first study, wild type and AT1R (type a) knockout mice were used. In the water and sodium depletion condition, with intakes expressed as ml/30 g body weight (the average weight of mice in the study), the water and 0.3 M NaCl 2 h test intakes (0.8 and 0.7 mL, respectively) observed in the wild type mice were attenuated (water) and abolished (salt) in the AT1R knockout group. In the sodium depletion condition, NaCl (0.9 mL) but no water was consumed by wild type mice, and this sodium appetite was completely absent in the knockout mice. Thus, in both conditions, salt intake after depletion was fully dependent on functional AT1 (type a) receptors, but their location was not known.

In the next experiment, Matsuda et al. [38] localized the critical receptors to the SFO. First, they found substantial expression of Fos-ir in SFO and OVLT, but not AP or PVN, after sodium depletion and, using a marker lacZ for AT1R-expressing neurons, showed that 82% and 65% of the Fos-expressing cells were AT1R-positive in SFO and OVLT, respectively. As expected, there was no corresponding Fos expression in AT1R knockout mice. They also found that Fos-ir was expressed in SFO and OVLT by water deprivation, but a smaller fraction of neurons was AT1R positive. Thus, salt and water deprivation engage different but overlapping populations of SFO and OVLT neurons. In this context, it is interesting to note that central but not peripheral administration of ANG II induces water intake in mice [44,45]. Given that peripherally administered ANG II is dipsogenic in rats and depends on an intact SFO, the source of the SFO AT1R stimulation by water deprivation in mice [38] should be considered an open question.

Next, to determine the relative contributions of the SFO and OVLT, mice with *loxP* flanking the AT1R gene were microinjected with Cre-AAV into one of these regions. This procedure causes selective loss of AT1R in either SFO or OVLT [38]. After sodium depletion, mice with injection into SFO showed a loss of NaCl intake that was proportional to the number of infected (Cre positive) cells. The group mean reduction was ~75%. In contrast, loss of AT1R in OVLT had no effect on sodium appetite. Thus, the sodium appetite after depletion in mice is completely dependent on ANG II signaling in the SFO. This is consistent with some but not all of the findings in rats with which we started this section.

Another region receiving glutamatergic neural projections from SFO is the ventral BNST. The neurons projecting from SFO to MnPO and ventral BNST are distinct but intermingled. Selective lesion or treatment of ventral BNST with a retrograde tracer and optically sensitive inhibitory channel in mice showed that sodium appetite is carried exclusively by this SFO-to-BNST excitatory (glutamatergic) pathway, and that water intake is not [38]. From in vitro studies, it was found that the excitatory SFO neurons projecting to ventral BNST may be modulated by GABAergic input arising from Na_x_ channels on glial cells in the SFO. In summary, Matsuda et al. [38] showed that sodium appetite after furosemide depletion in mice is entirely dependent on an ANG II-activated pathway from SFO to ventral BNST.

### 2.4. Integration of Brain Systems

We mentioned previously that a set of neurons in or anterior to the LC received projections from the HSD2-cells in the NST [34,36]. Geerling’s group [46] extended this analysis and further compared rats and mice. While the neurons activated in rats are clearly rostral to the LC (i.e., pre-LC), in mice they are mingled with LC neurons. They propose that the subset of cells in this region and in the central lateral PBN relevant to sodium homeostasis in rats have three properties. First, they exhibit Fos-ir following sodium depletion. Second, they express FoxP2. Third, they are within a region that receives ascending axons from NST and descending axons from the PVN. They do not overlap with catecholaminergic cells of the LC. In mice, the pre-LC cells are also distinct from catecholamine cells but are comingled.

Lee et al. [47] showed, in mice, that cells in the pre-LC that express Fos-ir after sodium depletion also are glutamatergic and co-express prodynorphin. By transfecting these excitatory dynorphin cells with ChR2, it was shown that optical stimulation of the pre-LC caused vigorous licking for 0.5 M NaCl solution and rock salt. Conversely, using an inhibitory (chloride channel) form of channelrhodopsin, sodium intake after sodium depletion was inhibited by 75% by photoinhibition of the pre-LC dynorphin cells. Other experiments conducted by these authors [47] showed that photostimulation of the peri-LC cells produces an aversive state. Examination of the real-time activity of these pre-LC cells using a fluorescent calcium indicator (GCaMP6s) showed that sodium ingestion by depleted mice suppressed the activity of these cells almost immediately, and even very brief access to salt (1 s) suppressed the calcium signal for several minutes. These effects of the ingestion of sodium were blocked by amiloride, indicating that gustatory information, and specifically sodium, suppresses the activity in pre-LC. Further, and consistent with results of Wolf, Schulkin & Simson [12] in rats, intragastric repletion with NaCl did not inhibit these neurons in the short term. These authors also showed that pre-LC cells receive monosynaptic inhibitory input from dynorphin-expressing cells in the dorsal BNST. Using the calcium reporter, they showed that the dynorphin-expressing cells in dorsal BNST are activated by sodium ingestion. Thus, the dorsal BNST appears to be the source of inhibition of pre-LC linked to the specific taste of sodium. This circuit would then have properties consistent with satiation of sodium appetite. However, as we noted earlier, rats and mice do not show appropriate satiation (i.e., they overconsume) when offered concentrated solutions of NaCl, so the quantitative aspects of operation of this proposed system remain unexplained.

The LPB is a heterogeneous structure that has also been implicated in ingestive behaviors, including inhibition of sodium appetite via a serotonergic mechanism in rats (e.g., [48,49]. More recently, Park and colleagues [50] extended and confirmed this analysis in mice. They found that sodium depletion, either by seven days of SDD or the furosemide-accelerated version, induced strong Fos-ir in the LPB in cells that express the serotonin 5HT2c receptor. When excited chemogenetically via a transfected HM3Dq receptor introduced into 5HT2c receptor-expressing cells in the LPB, sodium appetite in the furosemide-SDD protocol was inhibited by 50–70% over a 4-h test after injection of CNO. These cells, which receive serotonergic input from the raphe nuclei, send glutamatergic projections to several forebrain areas. The densest region of functional monosynaptic connections was the CeA. Injection of channel rhodopsin into 5HT2c-expressing cells of the LPB with subsequent photostimulation of the CeA suppressed sodium appetite by approximately 50% in sodium-depleted mice. Park et al. [50] interpret these and other findings in their paper as evidence for the chronic inhibition of sodium appetite by this LPB mechanism. Sodium appetite only occurs under conditions of depletion, so we see no good rationale to invoke a chronic inhibitory mechanism. Instead, what was confirmed was an inhibitory mechanism on induced appetite.

## 3. Summary and Conclusions

I have presented a restricted set of stimuli of sodium appetite in animals, described some older findings that guided the discovery of the mechanism, and present recent molecular biological approaches that have allowed a finer dissection of cell phenotypes and subnuclei involved in the induction of appetite and its satiation. The satiation mechanism is imperfect, featuring overconsumption in SDD-furosemide protocols [11] and Table 3). It is recommended that future studies of satiation emulate nature by using either SDD only, a sodium-rich food ([10] and Table 1), or an operant task.

## Figures and Tables

**Table 1:** 2 hr preference for salty food in sodium-depleted and non-depleted rats ^a^.

Salt Level ^b^	Depleted	Non-Depleted
0.5%	4.2 (78%) ^c^	1.2 (37%)
1%	3.8 (79%)	1.2 (40%)
2%	3.1 (82%)	0.8 (29%)
4%	2.0 (67%)	0.8 (25%)
8%	1.8 (60%)	0.1 (4%)

^a^ Data derived from Figure 3 of Bertino & Tordoff [10]. ^b^ Amount by weight NaCl added to sodium deficient diet (SDD). ^c^ Each cell shows 2 h intake (g) of salted food and as % of intake in a choice with SDD.

**Table 2:** Temporal changes of physiological parameters and sodium appetite following acute sodium depletion in rats ^a^.

Time (h) Since Furosemide	0 ^b^	3	12	24
Plasma aldosterone (pg/mL)	220	510 *	1200 *	1050 *
Plasma renin activity (ng AI/mL/h)	1	33 *	14 *	10 *
Plasma protein (g/dL)	8.6	9.2 *	9.0 *	8.8
Fos-ir in SFO (max. cells/section)	0	220 *	140 *	200 *
Fos-ir in OVLT (max. cells/section)	0	125 *	60 *	150 *
Intake (ml) 0.3M NaCl in 1 h	1.5	4.0 *	6.0 *	10.0 *

^a^ Data from Rowland & Morian [16]. ^b^ All rats were fed sodium deficient diet (SDD) for 24 h before furosemide. Time 0 groups were studied at this time but without injection of furosemide. * Significantly higher than time 0.

**Table 3:** Sodium intake and balance during first hour test conducted 24 h after furosemide or vehicle treatment with differential prior dietary exposure ^a^.

	Treatment Group			
	Chow-Vehicle	SDD-Vehicle	Chow-Furosemide	SDD-Furosemide
Initial sodium balance (mEq)	−1.0	0	−2.6	−1.5
Initial Urinary K/Na	2	70	16	45
1 h intake (mEq) 0.51 M NaCl	0.2	0.5	1.5	3.5
Sodium balance @ 1 h (mEq)	−0.8	0.5	−1.1	2.0

^a^ Data from Figures 2 and 4 of [11]. Designated diet was presented for 6 days beforehand.

## Data Availability

Not applicable.

## Abbreviations

AAV	Adeno-associated virus
ACTH	Adrenocorticotrophic hormone
ADH	Antidiuretic hormone
ANG II	Angiotensin II
AP	Area postrema
AT1R	Angiotensin II type 1 receptor
BNST	Bed nucleus of the stria terminalis
CeA	Central nucleus of amygdala
CNO	Clozapine-N-oxide
ECF	Extracellular fluid
EPSP	Excitatory postsynaptic potential
Fos-ir	Fos-like immunoreactivity
HSD2	Hydroxysteroid dehydrogenase type 2
ICF	Intracellular fluid
LC	Locus coeruleus
MnPO	Median preoptic nucleus
NST	Nucleus of the solitary tract
OVLT	Organum vasculosum of the lamina terminalis
PBN	Parabrachial nucleus
PRA	Plasma renin activity
PVN	Paraventricular nucleus of the hypothalamus
SDD	Sodium deficient diet
SFO	Subfornical organ
